# Complementary value of molecular, phenotypic, and functional aging biomarkers in dementia prediction

**DOI:** 10.1007/s11357-024-01376-w

**Published:** 2024-10-24

**Authors:** Andreas Engvig, Karl Trygve Kalleberg, Lars T. Westlye, Esten Høyland Leonardsen, Alzheimer’s Disease Neuroimaging Initiative Consortium, Michael Weiner, Paul Aisen, Ronald Petersen, Clifford R. Jack, William Jagust, John Q. Trojanowski, Arthur W. Toga, Laurel Beckett, Robert C. Green, Andrew J. Saykin, John C. Morris, Leslie M. Shaw, Enchi Liu, Tom Montine, Ronald G. Thomas, Michael Donohue, Sarah Walter, Devon Gessert, Tamie Sather, Gus Jiminez, Danielle Harvey, Matthew Bernstein, Nick Fox, Paul Thompson, Norbert Schuff, Charles DeCarli, Bret Borowski, Jeff Gunter, Matt Senjem, Prashanthi Vemuri, David Jones, Kejal Kantarci, Chad Ward, Robert A. Koeppe, Norm Foster, Eric M. Reiman, Kewei Chen, Chet Mathis, Susan Landau, Nigel J. Cairns, Erin Householder, Lisa Taylor Reinwald, Virginia Lee, Magdalena Korecka, Michal Figurski, Karen Crawford, Scott Neu, Tatiana M. Foroud, Steven Potkin, Li Shen, Faber Kelley, Sungeun Kim, Kwangsik Nho, Zaven Kachaturian, Richard Frank, Peter J. Snyder, Susan Molchan, Jeffrey Kaye, Joseph Quinn, Betty Lind, Raina Carter, Sara Dolen, Lon S. Schneider, Sonia Pawluczyk, Mauricio Becerra, Liberty Teodoro, Bryan M. Spann, James Brewer, Helen Vanderswag, Adam Fleisher, Judith L. Heidebrink, Joanne L. Lord, Ronald Petersen, Sara S. Mason, Colleen S. Albers, David Knopman, Kris Johnson, Rachelle S. Doody, Javier Villanueva Meyer, Munir Chowdhury, Susan Rountree, Mimi Dang, Yaakov Stern, Lawrence S. Honig, Karen L. Bell, Beau Ances, Maria Carroll, Sue Leon, Erin Householder, Mark A. Mintun, Stacy Schneider, Angela Oliver, Randall Griffith, David Clark, David Geldmacher, John Brockington, Erik Roberson, Hillel Grossman, Effie Mitsis, Leyla deToledo-Morrell, Raj C. Shah, Ranjan Duara, Daniel Varon, Maria T. Greig, Peggy Roberts, Marilyn Albert, Chiadi Onyike, Daniel D’Agostino, Stephanie Kielb, James E. Galvin, Dana M. Pogorele, Brittany Cerbone, Christina A. Michel, Henry Rusinek, Mony J. de Leon, Lidia Glodzik, Susan De Santi, P. Murali Doraiswamy, Jeffrey R. Petrella, Terence Z. Wong, Steven E. Arnold, Jason H. Karlawish, David A. Wolk, Charles D. Smith, Greg Jicha, Peter Hardy, Partha Sinha, Elizabeth Oates, Gary Conrad, Oscar L. Lopez, MaryAnn Oakley, Donna M. Simpson, Anton P. Porsteinsson, Bonnie S. Goldstein, Kim Martin, Kelly M. Makino, M. Saleem Ismail, Connie Brand, Ruth A. Mulnard, Gaby Thai, Catherine McAdams-Ortiz, Kyle Womack, Dana Mathews, Mary Quiceno, Ramon Diaz Arrastia, Richard King, Myron Weiner, Kristen Martin Cook, Michael DeVous, Allan I. Levey, James J. Lah, Janet S. Cellar, Jeffrey M. Burns, Heather S. Anderson, Russell H. Swerdlow, Liana Apostolova, Kathleen Tingus, Ellen Woo, Daniel H. S. Silverman, Po H. Lu, George Bartzokis, Neill R. Graff Radford, Francine Parfitt, Tracy Kendall, Heather Johnson, Martin R. Farlow, Ann Marie Hake, Brandy R. Matthews, Scott Herring, Cynthia Hunt, Christopher H. van Dyck, Richard E. Carson, Martha G. MacAvoy, Howard Chertkow, Howard Bergman, Chris Hosein, Sandra Black, Bojana Stefanovic, Curtis Caldwell, Ging Yuek Robin Hsiung, Howard Feldman, Benita Mudge, Michele Assaly Past, Andrew Kertesz, John Rogers, Dick Trost, Charles Bernick, Donna Munic, Diana Kerwin, Marek Marsel Mesulam, Kristine Lipowski, Chuang Kuo Wu, Nancy Johnson, Carl Sadowsky, Walter Martinez, Teresa Villena, Raymond Scott Turner, Kathleen Johnson, Brigid Reynolds, Reisa A. Sperling, Keith A. Johnson, Gad Marshall, Meghan Frey, Jerome Yesavage, Joy L. Taylor, Barton Lane, Allyson Rosen, Jared Tinklenberg, Marwan N. Sabbagh, Christine M. Belden, Sandra A. Jacobson, Sherye A. Sirrel, Neil Kowall, Ronald Killiany, Andrew E. Budson, Alexander Norbash, Patricia Lynn Johnson, Thomas O. Obisesan, Saba Wolday, Joanne Allard, Alan Lerner, Paula Ogrocki, Leon Hudson, Evan Fletcher, Owen Carmichael, John Olichney, Charles DeCarli, Smita Kittur, Michael Borrie, T. Y. Lee, Rob Bartha, Sterling Johnson, Sanjay Asthana, Cynthia M. Carlsson, Steven G. Potkin, Adrian Preda, Dana Nguyen, Pierre Tariot, Adam Fleisher, Stephanie Reeder, Vernice Bates, Horacio Capote, Michelle Rainka, Douglas W. Scharre, Maria Kataki, Anahita Adeli, Earl A. Zimmerman, Dzintra Celmins, Alice D. Brown, Godfrey D. Pearlson, Karen Blank, Karen Anderson, Robert B. Santulli, Tamar J. Kitzmiller, Eben S. Schwartz, Kaycee M. Sink, Jeff D. Williamson, Pradeep Garg, Franklin Watkins, Brian R. Ott, Henry Querfurth, Geoffrey Tremont, Stephen Salloway, Paul Malloy, Stephen Correia, Howard J. Rosen, Bruce L. Miller, Jacobo Mintzer, Kenneth Spicer, David Bachman, Elizabether Finger, Stephen Pasternak, Irina Rachinsky, John Rogers, Andrew Kertesz, Dick Drost, Nunzio Pomara, Raymundo Hernando, Antero Sarrael, Susan K. Schultz, Laura L. Boles Ponto, Hyungsub Shim, Karen Elizabeth Smith, Norman Relkin, Gloria Chaing, Lisa Raudin, Amanda Smith, Kristin Fargher, Balebail Ashok Raj

**Affiliations:** 1 Department of Endocrinology, Obesity and Preventive Medicine, Section of Preventive Cardiology, Oslo University Hospital, Oslo, Norway; 2 Age Labs AS, Oslo, Norway; 3 Department of Psychology, University of Oslo, Oslo, Norway; 4 Centre for Precision Psychiatry, Oslo University Hospital & Institute of Clinical Medicine, University of Oslo, Oslo, Norway

**Keywords:** Biological age, Brain age, Dementia, Methylation age, Frailty index, Deep learning, Machine learning

## Abstract

DNA methylation age (MA), brain age (BA), and frailty index (FI) are putative aging biomarkers linked to dementia risk. We investigated their relationship and combined potential for prediction of cognitive impairment and future dementia risk using the ADNI database. Of several MA algorithms, DunedinPACE and GrimAge2, associated with memory, were combined in a composite MA alongside BA and a data-driven FI in predictive analyses. Pairwise correlations between age- and sex-adjusted measures for MA (aMA), aBA, and aFI were low. FI outperformed BA and MA in all diagnostic tasks. A model including age, sex, and aFI achieved an area under the curve (AUC) of 0.94 for differentiating cognitively normal controls (CN) from dementia patients in a held-out test set. When combined with clinical biomarkers (apolipoprotein E ε4 allele count, memory, executive function), a model including aBA and aFI predicted 5-year dementia risk among MCI patients with an out-of-sample AUC of 0.88. In the prognostic model, BA and FI offered complementary value (both *β*s 0.50). The tested MAs did not improve predictions. Results were consistent across FI algorithms, with data-driven health deficit selection yielding the best performance. FI had a stronger adverse effect on prognosis in males, while BA’s impact was greater in females. Our findings highlight the complementary value of BA and FI in dementia prediction. The results support a multidimensional view of dementia, including an intertwined relationship between the biomarkers, sex, and prognosis. The tested MA’s limited contribution suggests caution in their use for individual risk assessment of dementia.

**Supplementary information:**

The online version contains supplementary material available at 10.1007/s11357-024-01376-w.

## Introduction

Dementia prevalence increases exponentially after age 65 [1], but the underlying biological mechanisms linking aging to dementia remain elusive [2]. One hypothesis holds that neurodegenerative diseases leading to dementia may be manifestations of accelerated aging [3]. By quantifying deviations in biological age from expected chronological age with aging biomarkers, we might identify individuals at higher dementia risk and assess the effects of interventions targeting aging and aging-related neurodegenerative processes [2, 3].

Various biomarkers of biological age or aging have shown promise in predicting a diagnosis of dementia or future risk of the disease [4–13]. Still, geroscience lacks a standard definition of biological aging and its ideal biomarker [14, 15]. The search for biomarkers is complex due to aging manifesting at multiple levels: molecular, phenotypic, and functional [16]. Here, we investigated the interrelated role of three leading aging biomarkers in dementia risk prediction, with each biomarker representing one of the three levels proposed by Ferruci et al. [16].

At the molecular level, alterations in methylation patterns of DNA are a hallmark of aging [17]. The methylation states in CpG dinucleotides across the genome can be measured in peripheral blood [18]. Algorithms called epigenetic clocks leverage this data to calculate an individual’s epigenetic or methylation age (MA). Often, MA is statistically adjusted for chronological age to generate an “age acceleration” metric [2], which quantifies the deviation of an individual’s methylation pattern from what is expected given their chronological age. Here, positive values indicate that an individual’s biological age as reflected by MA is higher than their chronological age. Several studies have used epigenetic age acceleration to study dementia risk [9, 10, 19–23]. For instance, Mcmurran et al. found that epigenetic age acceleration, as measured by the Horvath and GrimAge MAs increases future dementia risk [21]. Another study found that an epigenetic clock reflecting aging pace (DunedinPACE) was associated with current cognitive impairment and future dementia risk [9, 10]. Other studies, including a systematic review, have yielded mixed results [19, 22, 23] and highlight the need for further research. While single-gene methylation states were shown to predict a diagnosis of dementia due to Alzheimer’s disease (AD) with high in-sample accuracy in one study [24], we are unaware of studies examining the predictive power of MA algorithms for individual dementia risk assessment based on out-of-sample verification. In contrast, the predictive potential of phenotypic biomarkers, in particular those captured by brain age models, has been more frequently examined in the context of individual risk assessment [7, 25, 26].

A notable example of a phenotypic aging biomarker is the difference between magnetic resonance brain imaging (MRI)-predicted and chronological age, denoted the brain age gap (BAG) [25, 27, 28]. The individual’s brain age (BA) is calculated by algorithms trained with machine learning, with recent BA models repeatedly producing reliable estimates across wide age ranges [27]. Studies have found positive associations between elevated BAG, AD biomarkers, and cognitive status [6, 13]. Importantly, BAG has also been tested for individual dementia prediction. For example, using BAG, Persson et al. achieved an area under the receiver operating characteristic curve (AUC) of 0.68 in classifying individuals with dementia from non-dementia [7].

Aging eventually manifests as functional deterioration [15, 16]. Functional aging is frequently quantified using a frailty index (FI) [29, 30]. FI is a composite score that reflects the accumulation of health deficits [29]. Multiple studies have demonstrated that a greater degree of frailty, as measured by higher FI scores, correlates positively with poorer cognitive status and future dementia risk [31], even independent of cognitive test results [8]. The latter study by Song et al. [8] obtained an AUC of 0.64 and 0.66 for the prediction of 5-year and 10-year future dementia risk.

A cross-sectional study by Phyo and colleagues [32] was the first to compare the three reviewed biomarkers representing different hierarchical levels of aging (MA, BAG, and FI). The authors found a mostly low correlation between MA measures and FI, while BAG was not correlated with either. The findings suggested a possible complementary value of the three in predicting age-related disease risk. While this has yet to be examined in the context of dementia, studies investigating another aging-related endpoint—mortality—offer support for this notion. Li and colleagues [33], as well as Li et al. [34], found that the combination of different epigenetic clocks and FI scores enhanced mortality risk prediction compared to using any of these biomarkers in isolation. Moreover, Cole et al. found that combining BAG and MA improved mortality risk prediction [35]. These findings highlight the potential power of combining biomarkers from different levels of aging to improve the prediction of age-related outcomes. To date, a direct comparison of molecular, phenotypic, and functional aging biomarkers in the context of dementia prediction is lacking.

Our primary objective was to assess the individual and combined predictive value of the reviewed aging biomarkers (measures of MA, BA, and FI) for current cognitive impairment and future dementia risk utilizing the Alzheimer’s Disease Neuroimaging Initiative (ADNI) database. We hypothesized that combining biomarkers from each of the hierarchical levels of aging would improve model performance compared to using them individually. Our secondary objective was to examine the predictive potential of the aging biomarkers when incorporated into models alongside well-established, accessible clinical biomarkers: number of apolipoprotein E ε4 (APOE4) alleles and cognitive tests (memory, executive function). We anticipated that the aging biomarkers would provide added predictive value beyond these clinical markers.

For both objectives, we employed machine learning classifiers with out-of-sample verification, using AUC as our main performance metric. Theoretically, functionally apparent aging emerges when resilience mechanisms at upstream levels become exhausted. Consequently, we hypothesized that “age acceleration” at the functional level (measured by FI) would be a stronger predictor of dementia-related outcomes compared to an upstream phenotypic measure (BA). In turn, we expected phenotypic age acceleration to be a stronger predictor than upstream molecular metrics. Finally, we expected sex disparities in the aging biomarkers [32] and conducted sensitivity analyses to evaluate sex differences in their predictive power.

## Methods

### Data source

Data used in the preparation of this article were obtained from the ADNI database (adni.loni.usc.edu). ADNI was launched in 2003 as a public–private partnership, led by Principal Investigator Michael W. Weiner, MD. The primary goal of ADNI has been to test whether serial MRI, positron emission tomography, other biological markers, and clinical and neuropsychological assessment can be combined to measure the progression of mild cognitive impairment (MCI) and early AD. For up-to-date information, see www.adni-info.org. For the purposes of the present study, we drew subjects from three observational prospective case–control ADNI cohorts called ADNI1, ADNI2, and ADNIGO. Eligible subjects had available brain MRI, DNA methylation, and FI data obtained within 90 days of the baseline visit; cognitive test results and demographics were drawn from the baseline visit. Data are publicly available (https://ida.loni.usc.edu/).

### Sample

Inclusion criteria included age 55 to 90 years; study partner to provide evaluation of function; speaks English; ability to undergo all testing, blood samples for genotyping and biomarkers, and neuroimaging procedures; completed six grades of education or work history; for women postmenopausal or surgically sterile, not depressed, and a modified Hachinski score less than five to rule out vascular dementia. Individuals with dementia (hereafter abbreviated “DEM”) satisfied the criteria for NINCDS/ADRDA for probable AD DEM. Importantly, we used the clinical diagnosis reported in the ADNI database and did not require Alzheimer’s disease pathology biomarkers. Subjects enrolled as MCI had memory complaints verified by a study partner, Mini Mental Status Examination (MMSE) score of 24 to 30, Clinical Dementia Rating (CDR) global score (CDR-GS) = 0.5 with sum of boxes (CDR-SB) score of at least 0.5, and general cognition and functional performance sufficiently preserved such that a diagnosis of DEM could not be made. AD biomarkers were not required for a diagnosis of MCI in the present study. Cognitively normal (CN) controls had MMSE scores of 24 to 30, CDR-GS = 0 (with CDR-SB score = 0), and were deemed normal based on an absence of significant impairment in cognitive functions or activities of daily living. In addition to the functional tests used to determine diagnosis, the participants underwent standard neuropsychological assessment at baseline, including Rey’s Auditory Verbal Learning Test (RAVLT) and the Trail Making Test (TMT) which probes hallmark cognitive subdomains (memory and executive function, respectively) associated with AD DEM [36–39]. For the present study, we selected the immediate recall part of RAVLT (simply denoted RAVLT throughout for brevity) for memory and part B of TMT (TMT-B) for executive function.

### Molecular age

We quantified biological age at the molecular level by evaluation of DNA methylation (DNAm) patterns in white blood cells obtained from peripheral blood samples. DNAm data profiling was previously performed by the ADNI investigators for 653 unique ADNI participants using the Illumina Infinium HumanMethylationEPIC BeadChip Array (www.illumina.com). We used a subset of these (*n* = 385) that also had available MRI and FI data within 90 days of the baseline visit. If more than one DNAm sample existed for each unique participant, we selected the sample from the baseline visit. If a baseline sample was not available, we drew the sample at the temporally closest time point to the baseline, excluding cases where the baseline-to-sample interval was 90 days or more apart. Median (absolute) time between baseline visit and blood sampling was 0.5 days (interquartile range (IQR) = 0 to 7) and was similar between diagnostic groups and stable vs progression MCI (Wilcoxon tests, *p*-values > 0.05).

Several state-of-the-art MA algorithms may be used to obtain measures of aging from blood cell DNAm data. Here, we selected seven candidate MA algorithms that have been associated with either neuropsychological test results, diagnosis, and/or future conversion to dementia risk in one or more observational studies [10, 18, 21–23, 40–43]: Horvath’s first-generation epigenetic clock (DNAmAge [44]), Horvath’s epigenetic “Skin-Blood” clock (Skin-BloodClock [45]), second generation PhenoAge [46], two iterations of GrimAge (i.e., GrimAge [41], GrimAge2 [40]), and PCBrainAge [43] epigenetic clocks, and a third generation pace of aging MA measure, DunedinPACE [47]. Given the lack of a single or gold standard MA algorithm for DEM prediction and the inconsistent association between present MA measures and neurocognitive outcomes, the best MA metrics were decided via exploratory data analysis in the training set prior to the predictive modelling (details provided below) and reduced to a MA composite measure (denoted MA) using principal component analysis (PCA). The composite MA was rank-transformed to obtain a similar distribution as FI and BA.

### Phenotypic age

We used BA to operationalize aging at the phenotypic level. We estimated BA using a previously published deep neural network that has been shown to generalize to unseen scanners and cohorts [48], and which is freely available online (https://github.com/estenhl/pyment-public). Briefly, this is a convolutional neural network with an architecture consisting of six blocks of convolutional and max-pooling layers. Prior to modelling, T1-weighted MRI scans were minimally processed to remove non-brain tissue with the recon-all pipeline from FreeSurfer 5.3. Further, they were rigidly registered to the same stereotactic space, using flirt from FSL with six degrees of freedom. Further details of the pipeline are described in the original publication [48]. In cases where participants had multiple MRI scans, we chose the imaging session closest to baseline and excluded cases where the baseline-to-scan interval was 90 days or more apart. The median (absolute) baseline to scan interval was 16 days (IQR = 10 to 27).

### Functional age

We operationalized aging at a functional level using an FI based on the accumulation of deficits model of frailty developed by Rockwood and Mitnitski [29]. We first manually selected 93 health deficits, including common clinical variables, available at the screening or baseline visit in the ADNI database, adhering to the standard procedure by Searle et al. [30]. Then, we refined the manual selection of deficits using a data-driven procedure that combines factor analysis of mixed data, cluster analysis, and regression analysis [5]. This refinement aimed to reduce the number of deficits while maximizing explained variance. The resulting data-driven FI consisted of 26 items and has previously been validated against mortality and DEM risk in ADNI and has been shown to be superior for DEM prediction compared to FIs generated using the standard procedure only [5]. In previous work, the data-driven FI employed here was denoted FI_r_ (*r* for refined), and a detailed description of its calculation, including items and cut-offs, has been published [5]. The code for calculating the present, data-driven FI using ADNI data is also freely available (https://github.com/LAMaglan/ADNI-FI-clustering). This FI variable contains health deficits covering a range of systems, including non-cognitive clinical tests, such as blood test results (red and white blood cell counts), blood pressure, history of disease, symptoms such as low energy, alterations in gait, and functional measures. We use the term FI below when addressing the abovementioned data-driven FI unless otherwise specified. While superior to DEM prediction in ADNI, the data-driven FI used herein might not be readily applicable to FIs in general, as the standard procedure does not involve an unsupervised machine-learning step for selecting health deficits [49]. To address this, we also ran our analysis-scheme using an FI constructed only using the standard procedure in supplementary analyses. For details regarding the included items and cut-offs used for generating the standard FI, refer to [50]. For both FIs, higher scores indicate a greater degree of frailty (i.e., lower level of physical or systems-level function).

### Age and sex-adjusted biomarkers

To derive comparable measures of aging at the three different levels, we first adjusted each measure by regressing out sex and chronological age using linear models. These were fit using the training data (see below) and then applied to both the training and test data to produce residuals instead of raw measures, representing sex- and age-adjusted values. Next, we standardized the residuals by subtracting the mean and dividing by the standard deviation to obtain *z*-scores for subsequent modelling. The deviations from expected values based on sex and chronological age at different levels (i.e., biological, phenotypic, and functional) were denoted by adding the prefix “a” to the adjusted measures: aFI, aBA, and aMA. The same age-adjustment and standardization procedure was done for RAVLT (aRAVLT) and TMT-B (aTMT-B) to render variants of these uncorrelated with sex and age. To explicitly assess the impact of chronological age and sex in the predictive models, we included these as predictors when appropriate.

### Statistical analyses

**Fig. 1:**
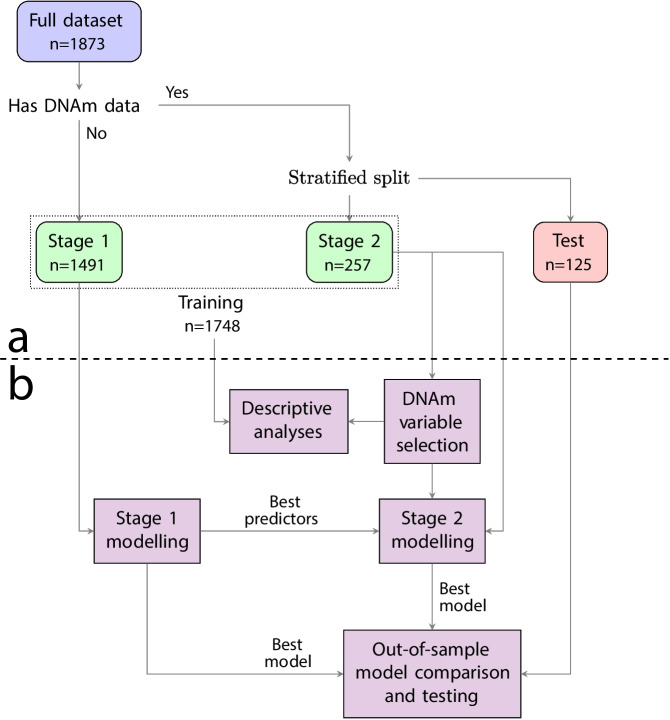
**a** The dataset was split into subsets to facilitate hyperparameter-tuning and obtain unbiased out-of-sample estimates of model performance. Due to various combinations of missing biomarker data, we first extracted all participants with complete frailty index (FI) and brain age (BA) data, but lacking methylation data into a stage 1 modelling training set (*n* = 1491). This was used to identify the optimal selection of age- and sex-adjusted BA and FI (i.e., aBA, aFI, or aBA + aFI) as predictors across all predictive tasks. The remaining 385 participants (with complete FI, BA, and DNA methylation data) were stratified using diagnosis, age, and sex and divided into another training set for a second modelling stage (stage 2) and a hold-out test set. **b** All training data was utilized for descriptive statistics, and we employed a data-driven variable selection procedure to determine which methylation age markers to use. After selecting the best combination of BA and FI as predictors, stage 2 modelling was done to find the optimal selection of all three aging biomarkers. The best models from stages 1 and 2 were compared in a cross-validation loop before evaluating the best model in the test set to obtain an unbiased estimate of model performance

Before any analyses were performed, we split the data into subsets to facilitate hyperparameter-tuning and unbiased out-of-sample estimates of model performance (Fig. 1). We aimed to use as much data as possible to fit the models, despite the participants having various combinations of the relevant measures. Starting from the full dataset (*n* = 1876), we first extracted all participants lacking methylation data into a stage 1 modelling dataset (*n* = 1491), to be used for identifying the optimal selection of BA and FI (e.g., only aBA, only aFI, or aBA + aFI) as predictors, across all predictive tasks. The remaining 385 participants (with DNAm data) were stratified using diagnosis, age, and sex before 260 were drawn to form the dataset for a second modelling stage (stage 2 dataset). The remaining 125 participants were reserved in a held-out test set.

**Fig. 2:**
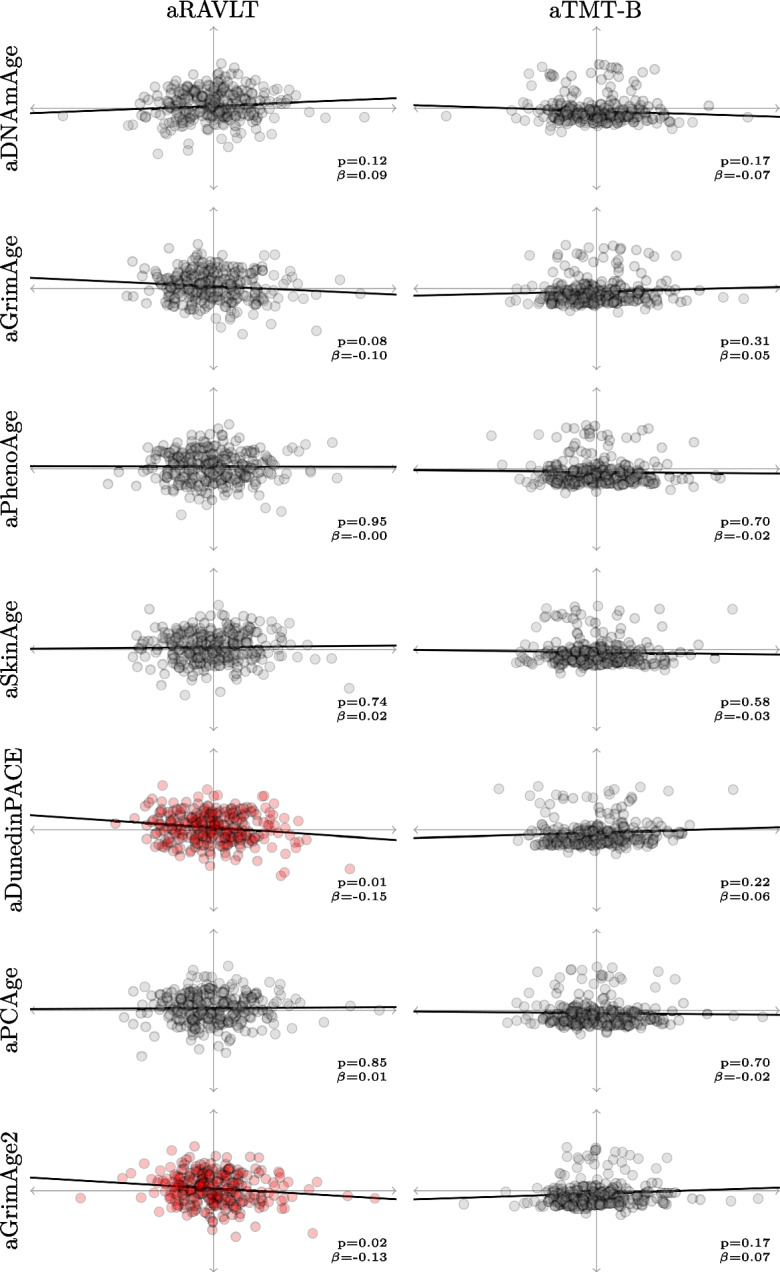
Scatterplots showing individual data and model fit lines from linear regressions from the stage 2 training dataset of age- and sex-adjusted candidate methylation ages (MA) and memory (Rey Auditory Verbal Learning Test, immediate recall (RAVLT), left column) and executive function (Trail Making Test, part B (TMT-B), right column). *β* represents the linear regression coefficient of each MA and the corresponding nominal *p*-value. Significant associations are colored

We performed data-driven feature selection in the stage 2 training dataset to identify a subset of candidate MA measures prior to any modelling. Here, we tested for associations between each of seven adjusted MA measures (aDNAmAge, aGrimAge, aPhenoAge, aSkin-BloodClock, aDunedinPACE, aPCBrainAge, aGrimAge2) and age- and sex-adjusted tests of memory (aRAVLT) and executive function (aTMT-B), which are well-known risk factors for DEM [36, 39]. We employed linear regression models for each univariate analysis (Fig. 2). Next, we retrieved the coefficient and *p*-value for each of these bivariate relationships from the models and corrected the *p*-values for multiple tests to reduce the false discovery rate (FDR) using the Benjamini–Hochberg procedure. Finally, MA measures with at least one *p*-value < 0.05 post-correction for any of the predictive targets were retained for the subsequent analyses. The adjusted MA measures that survived were combined into a single measure by fitting a PCA to them and retrieving the first principal component. As these measures were already adjusted for age and sex, this composite measure was left unadjusted (although we refer to it as aMA given the preceding adjustment steps).

**Fig. 3:**
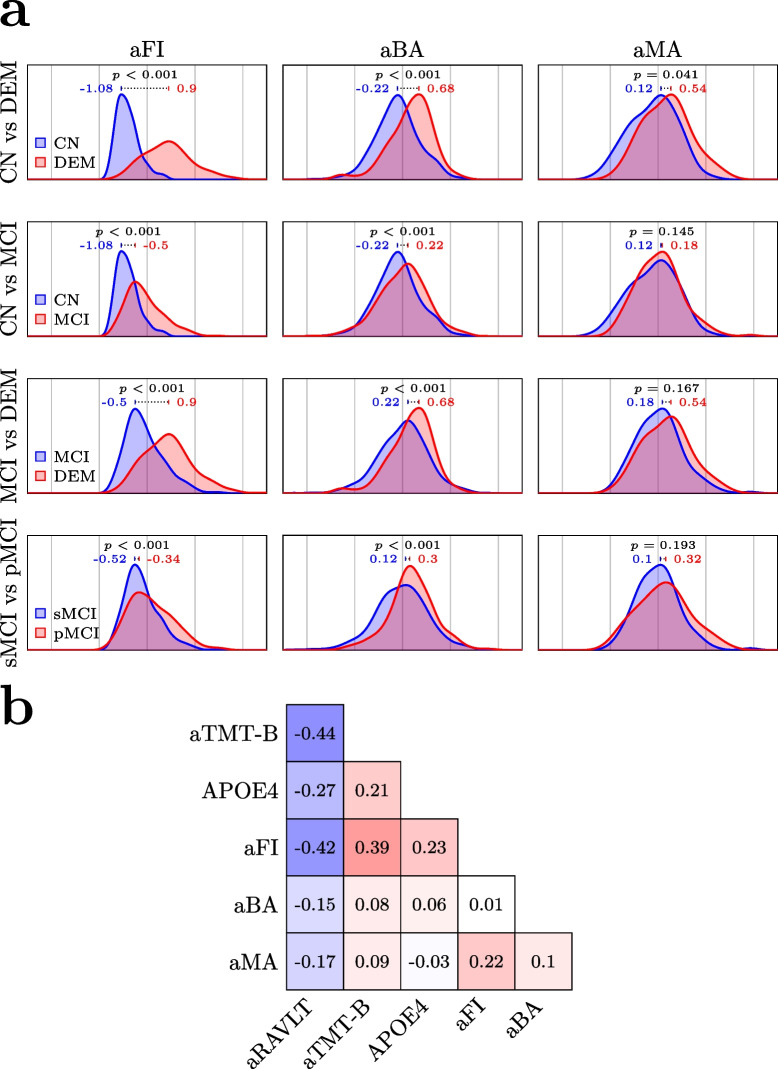
**a** Training set density plots illustrate the distributions of age- and sex-adjusted aging biomarkers (standardized residuals) in the subgroups used for diagnostic and prognostic classification. The 1st row shows the distributions for subjects with normal cognition (CN) and dementia (DEM), respectively, 2nd row for CN and subjects with mild cognitive impairment (MCI), 3rd row for MCI and DEM, and 4th row for subjects with MCI progressing to DEM within 5 years (pMCI) and subjects with MCI remaining stable (sMCI). Note: The results reported here are derived from all available training data for each of the biomarkers separately. As a result, medians and p-values shown in row 4 (sMCI, pMCI) differ slightly from those in Table 1 which are based on a smaller selection of subjects with complete data for all biomarkers. The blue and red values are subgroup aging biomarker medians. *p*-values are false discovery rate-adjusted from Kruskal–Wallis H-tests of subgroup differences in the biomarkers. **b** Pearson correlations between the adjusted aging biomarkers and common clinical markers in the training set. Additional abbreviations: prefix a = standardized age- and sex-adjusted residual; APOE, apolipoprotein E ε4 allele count; BA, brain age; FI, frailty index; MA, Composite methylation age; RAVLT, Rey Auditory Verbal Learning Test, immediate recall; TMT-B, Trail Making Test, part B

Next, we performed multiple descriptive analyses in the training set to assess the interrelationship between the aging biomarkers, and how they relate to the diagnostic and prognostic groups. Here, for prognostic comparison, we divided MCI subjects into those who converted to DEM within 5 years from baseline (progressive MCI, pMCI) and those who remained stable (stable MCI, sMCI). First, we investigated the distribution of values for each measure in the two groups comprising each of four binary partitions of the training set (Fig. 3a): CN vs DEM patients, CN vs MCI patients, DEM vs MCI patients, and sMCI vs pMCI, respectively. For each comparison, a Kruskal–Wallis H-test was employed to determine whether the medians of the two groups differed significantly, with *p*-values corrected to control the FDR. Lastly, we investigated the covariance between the measures, also in the stage 2 dataset, by computing all pairwise Pearson correlations (Fig. 3b). Here, we also included their correlation with aTMT-B, aRAVLT, and the number of APOE4 alleles (0, 1, or 2), coded as an ordinal variable.

We then fit logistic regression models to assess the predictive value of the aging biomarkers. These models were trained to optimize the standard log loss in combination with an ${l}_{1}$ regularization term. The latter was included for its variable selection properties to help substantiate our claims about the utility of our chosen predictors beyond the comparison of overall model performances. We first tested diagnostic predictions: For each of three predictive tasks (CN vs DEM, CN vs MCI, MCI vs DEM), this was performed in two stages, based on stages 1 and 2 datasets. First, we used stage 1 data to perform a ten-fold cross-validation (CV) to determine the best combination of aBA and aFI and the optimal value for the regularization parameter $\lambda$, fitting multiple models for each permutation of these two. The models were split while stratifying on diagnosis, progression, sex, and age. Each of the models included age and sex as additional predictors, and we also fit a fourth model containing only age and sex as a baseline. The best stage 1 model was determined as the one yielding the highest mean AUC across the ten validations. We used AUCs rounded to two decimals, and in the case of ties, we selected the simplest model (i.e., the one with the fewest predictors). Next, we performed a similar ten-fold cross-validation (CV) using the stage 2 data to determine whether to include aMA and the ideal value of $\lambda$. Here, we fit two models: one containing only age and sex as predictors alongside aMA, and one also including the best stage 1 predictors. As above, we selected the model yielding the mean CV AUC. Finally, we calculated the AUC of the best-performing model in the held-out test set.

In the final analysis, we investigated whether any of the three aging biomarkers at baseline provided prognostic value for detecting which MCI patients would progress into DEM within 5 years by differentiating the sMCI and pMCI cohorts. We deemed a 5-year timeframe to be clinically relevant while ensuring enough converting subjects for modelling. First, we performed the same variable selection process as above to identify the best set of predictors among the aging biomarkers. Next, we fit a model with clinical biomarkers, i..e., aTMT-B, aRAVLT, and APOE4, using the same training data as the candidate model chosen for the aging biomarkers (i.e., stages 1 or 2 data depending on the chosen predictors). Finally, we fit a model containing the best set of aging biomarker measures alongside the clinical, still using the same data. To investigate whether the two sets of covariates (aging, clinical) were complementary, we triangulated the AUCs achieved in the test set between these three models. For interpretability, and to allow for comparisons with other studies, we calculated the balanced accuracy, sensitivity, specificity, and positive predictive value of the best-performing model alongside the AUC. Given the ${l}_{1}$-loss employed attempting to nullify the coefficients of predictors not contributing to the predictions, we also report the non-zero coefficients used in the best-performing model to emphasize which variables contributed to the prediction.

To tease apart potential sex differences in the interrelatedness and predictive power of the proposed biomarkers, we supplemented the main prediction analyses with a similar analysis while splitting the dataset based on sex. Here, we fit models independently for males and females, keeping the standardized aging biomarkers and chronological age as predictors. Due to the smaller sample size resulting from analyzing male and female sub-groups separately, we compared the sex-specific results using 100 bootstrapped training and test subsets to yield distributions of both performance metrics and coefficient estimates.

## Results

### Descriptive statistics

The training dataset consisted of 1748 subjects (Fig. 1). A subset of 257 subjects had DNAm data obtained within 3 months of baseline and was used for stage 2 modelling. Within this subset, 157 had a baseline diagnosis of MCI with complete aging biomarker data where 26% converted to DEM within 5 years (Table 1).

**Table 1:** Baseline characteristics of mild cognitive impairment (MCI) subgroups based on 5-year dementia conversion status in training sample with complete biomarker data (*N* = 157)

	Conversion status		
Variables	Stable, *N* = 116	Progression, *N* = 41	*p*-value
Age, years			0.41
Median (IQR)	70.5 (66.2, 76.4)	72.7 (68.4, 76.1)	
Sex			0.70
Female	54 (47%)	17 (41%)	
Male	62 (53%)	22 (59%)	
MMSE, score			0.001
Median (IQR)	29.0 (28.0, 29.0)	27.0 (26.0, 29.0)	
APOE4, number of alleles			< 0.001
0	78 (67%)	14 (34%)	
1	30 (26%)	19 (46%)	
2	8 (6.9%)	8 (20%)	
aRAVLT (*z*-score)			< 0.001
Median (IQR)	0.2 (− 0.4, 0.7)	− 0.5 (− 1.0, − 0.2)	
aTMT-B (*z*-score)			0.02
Median (IQR)	− 0.4 (− 0.7, 0.0)	− 0.1 (− 0.5, 0.3)	
aDunedinPACE (*z*-score)			0.55
Median (IQR)	− 0.1 (− 0.5, 0.5)	0.3 (− 0.9, 0.7)	
aGrimAge2 (*z*-score)			0.09
Median (IQR)	− 0.1 (− 0.7, 0.4)	0.1 (− 0.4, 0.6)	
aMA (*z*-score)			0.23
Median (IQR)	0.0 (− 0.7, 0.5)	0.3 (− 0.7, 0.8)	
aBA (*z*-score)			0.15
Median (IQR)	− 0.1 (− 0.8, 0.6)	0.2 (− 0.2, 0.5)	
aFI (*z*-score)			< 0.001
Median (IQR)	− 0.2 (− 0.7, 0.2)	0.3 (− 0.2, 1.0)	

The table shows distributions of the proposed baseline predictors in progressive (pMCI) and stable (sMCI) MCI subgroups with complete biomarker data in the training set. pMCI subjects progressed to dementia within 5 years, while sMCI remained stable. *p*-values were calculated using the chi-squared test for APOE4 and sex and the Wilcoxon rank sum test for the remaining. Additional abbreviations: *APOE4* apolipoprotein E ε4. *MMSE* Mini Mental State Exam. Prefix “a” means that the biomarker is reported as the standardized age- and sex-adjusted residual (i.e., *z*-score) from linear regressions. *RAVLT* Rey’s Auditory Verbal Learning Test, Immediate recall score, *TMT-B* Trail Making Test, part B, *MA* methylation age (represented here as the 1st principal component from principal component analysis of GrimAge2 and DunedinPACE epigenetic clock variables), *BA* brain age, *FI* frailty index

Figure 2 shows correlations between adjusted MA measures and tests of memory and executive function. None of the 14 correlations remained significant after *p*-value correction (all adjusted *p*-values exceeded the alpha threshold of 0.05). Thus, we resolved to nominal *p*-values for variable selection, which yielded two significant correlations: Both increased aDunedinPACE and aGrimAge2 associated with poorer memory, i.e., lower scores on aRAVLT ($\beta$= − 0.15, nominal *p*-value = 0.01 and $\beta$ =  − 0.13, nominal *p*-value = 0.02, respectively). We retained aDunedinPACE and aGrimAge2 as our MA measures and combined them into a single measure, aMA, through PCA (Supplementary Fig. 1).

Figure 3a shows distributions of the adjusted aging biomarkers, aMA, aBA, and aFI in four binary partitions of the training set, each consisting of two disjunct groups (CN vs MCI, CN vs DEM, MCI vs DEM, sMCI vs pMCI). Here, all three measures showed a significant difference between the DEM patients and CN after FDR-correction: aFI was the largest (1.98 difference between group medians, adjusted *p* < 0.001), followed by aBA (0.9 difference, adjusted *p* < 0.001) and aMA (0.42 difference, adjusted *p* = 0.041). For the remaining three partitions, both aFI and aBA showed significant group differences in the expected direction, although with somewhat smaller differences between the groups than in the first partition, whereas aMA did not.

Figure 3b shows bivariate correlations for the adjusted aging biomarkers, executive and memory function, and APOE4 status in the training sample. Besides aRAVLT which is coded in the opposite direction (i.e., lower values mean more severe impairment), all measures except APOE4 and aMA were positively correlated, indicating general agreement. The highest absolute correlation was observed between aTMT-B and aRAVLT (− 0.44). The highest correlation containing at least one aging biomarker was between aFI and aRAVLT (− 0.42), indicating worse memory performance with an increasing degree of frailty. Among the aging biomarkers, the highest correlation was observed between aMA and aFI (0.22). Although there was concordance in the directionality among the three aging biomarkers (all positive values), their correlation was low (0.01 and 0.1 in addition to the 0.22 above), underscoring the possibility that they are sensitive to complementary information. Supplemental Figure 2 shows correlations among age-adjusted biomarkers for males and females separately.

### Predictive analyses: FI outperforms and renders BA and MA redundant in diagnostic group predictions

**Fig. 4:**
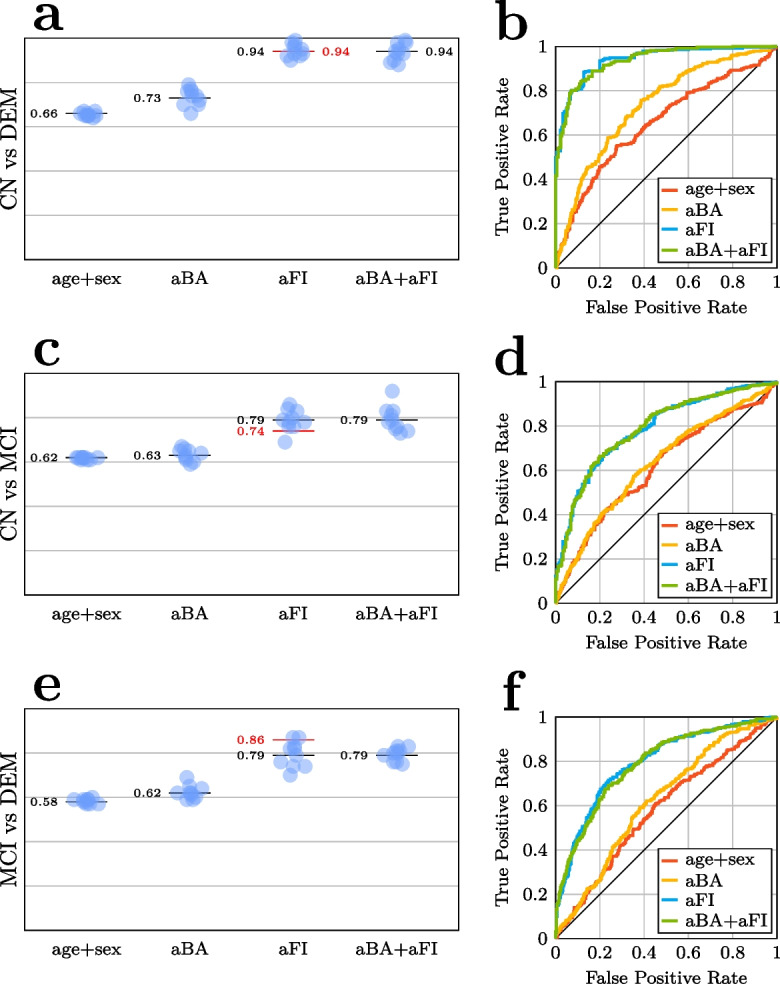
Predictive performance––reported as the area under the receiver operating characteristic curve (AUC)––for the various models for each of the three predictive (diagnostic) tasks. For each task, a baseline model was first fit in the stage 1 dataset using age and sex as predictors. Next, models including standardized age- and sex-adjusted brain age (aBA) and frailty index (FI) residuals, both independently and in combination, were trained using the same data. The *x*-axis denotes these different models, and the *y*-axis denotes their AUC. Individual points (blue) represent performance in independent folds in the cross-validation, whereas the black line denotes their mean. The red line represents the performance of the best model in the hold-out test set. For each row, the ROC curves underlying the AUC computation are shown on the right. Additional abbreviations: CN, cognitively normal; MCI, mild cognitive impairment; DEM, dementia

In predictive analyses, we first utilized the adjusted aging biomarkers as predictors in three binary diagnostic classification problems: CN vs DEM, CN vs MCI, and DEM vs MCI. Figure 4 shows model performance and receiver operating characteristic (ROC) curves for all problems in the stage 1 dataset. For CN vs DEM (Fig. 4a), the model containing only aBA, age, and sex outperformed a model containing age and sex only (mean CV AUC = 0.73 vs 0.66, respectively). However, this performance was greatly surpassed by the model containing aFI (CV AUC = 0.94), which was not improved upon by the one combining aBA and aFI (also CV AUC = 0.94). In the stage 2 dataset (Supplementary Fig. 3a), the model with aMA also outperformed the baseline model with age and sex (CV AUC = 0.64 vs 0.58, respectively); however, combining aMA with aFI did not outperform the model containing only aFI (both CV AUC = 0.98). The results indicate that neither aBA nor aMA complemented aFI in distinguishing CN and DEM patients. In a final test of model efficacy, the best model (i.e., the one with only aFI, age, and sex as predictors) achieved an out-of-sample AUC of 0.94 in the held-out test set.

For the classification of CN vs MCI (Fig. 4c), the best stage 1 model also contained only aFI (CV AUC = 0.79). As in the previous task, the best model from stage 1 (stage 2 CV AUC = 0.82, Supplementary Fig. 3c) was equivalent to the model including aMA (stage 2 CV AUC = 0.82), depicting that neither here did aBA or aMA add predictive value on top of aFI. For this task, the best model (once again containing only aFI) achieved an out-of-sample AUC of 0.74 in the held-out test set.

Also for MCI vs DEM (Fig. 4e), the best stage 1 model included only aFI (CV AUC = 0.79), although here, the model with aBA substantially outperformed the baseline model (CV AUC = 0.62 vs 0.58). Once again, the model including aMA did not improve upon the model with aFI in the stage 2 data (CV AUC = 0.81 for only aFI, 0.79 for aFI + aMA, Supplementary Fig. 3e). Retaining the model with only aFI as a predictor yielded an out-of-sample AUC of 0.86.

To summarize, the best models for differentiating all diagnostic groups included aFI as the sole aging biomarker, leaving aMA and aBA redundant for these tasks. For CN vs DEM, the results suggested an outstanding ability of aFI, age, and sex to differentiate between individuals living with DEM and healthy controls (out-of-sample AUC = 0.94), whereas acceptable and good performance was seen for CN vs MCI (out-of-sample AUC = 0.74) and MCI vs DEM (out-of-sample AUC = 0.86), respectively. Supplementary Figure 4a–c shows confusion matrices for these three predictive tasks.

### BA and FI complement each other and common clinical markers in the prediction of prognosis

**Fig. 5:**
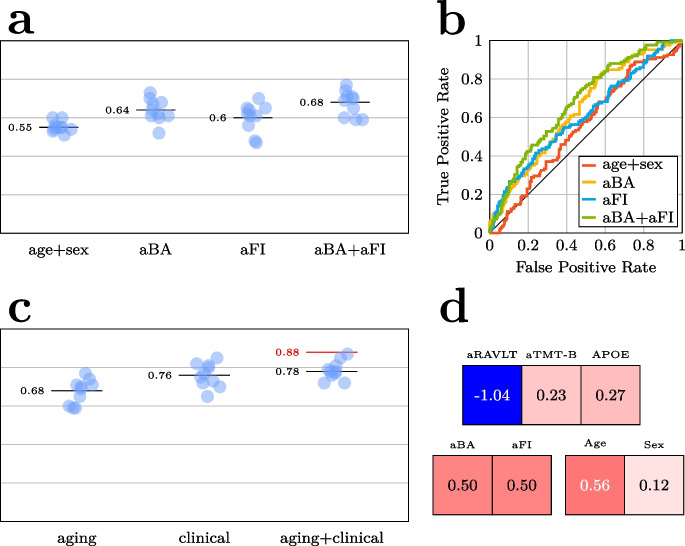
**a** Comparison of the prognostic models containing different subsets of the aging biomarkers for predicting progression from mild cognitive impairment (MCI) at baseline to dementia (DEM) within 5 years. **b** ROC curves underlying the AUCs computed in **a**. **c** Comparison of the best prognostic aging model with a prognostic model containing tests commonly used in the clinical workup of suspected DEM: apolipoprotein E ε4 allele count (APOE), the Rey Auditory Verbal Learning Test, immediate recall (RAVLT), and the Trail Making Test, part B (TMT-B). The final model denoted aging + clinical combines predictors from these two. All models included age and sex. For **a** and **c**, the individual points (blue) denote the area under the receiver operating characteristic curve (AUC) in independent folds in the cross-validation (CV), the black line is the CV mean, and the red line is the model performance of the best model in the hold-out test set. **d** The regression coefficients of the best-performing model for predicting 5-year DEM progression

Next, we investigated the efficacy of the aging biomarkers to differentiate sMCI and pMCI patients, effectively attempting to answer the clinical question “Will this patient with MCI convert to DEM over the next 5 years?” Here, the best stage 1 model included both aFI and aBA (CV AUC = 0.68, Fig. 5a), with both variables bringing substantial and similar predictive gains (CV AUC = 0.64 for only aBA, 0.6 for only aFI, vs 0.55 for the model including age and sex only). Supplementary Figure 3 shows that a model containing aMA on top of aBA and aFI failed to yield further predictive gains in the stage 2 CV (AUC = 0.72 for aBA + aFI, compared to 0.7 for aMA + aBA + aFI). We thus retained the model with aFI and aBA.

To establish a baseline for determining whether the aging biomarkers aBA and aFI provide additional prognostic value beyond predictors commonly used in dementia work-up, we trained a model using age, sex, and clinical test results (aRAVLT, aTMT-B, and APOE4 allele count), yielding a mean CV AUC of 0.76 (Fig. 5c). As shown in Fig. 5c, we trained another model that included age, sex, the clinical tests, and aFI and aBA as aging biomarkers, yielding a mean CV AUC of 0.78, indicating acceptable classification performance. Importantly, this result indicates that the enhanced model incorporating the aging biomarkers outperformed the one with clinical markers by themselves. This final model reached an AUC of 0.88 in the held-out test set, corresponding to good discrimination between individuals with MCI progression to dementia within 5 years and those who remain stable. In the test set, the enhanced model had a balanced accuracy of 77.25%, sensitivity of 0.85, specificity of 0.69, and positive predictive value of 0.49. Supplementary Figure 4d shows the model’s confusion matrix. Despite the ${l}_{1}$-loss attempting to eliminate superfluous covariates, all coefficients remained non-zero (Fig. 5d), with aRAVLT having the largest absolute value (− 1.01), followed by age (0.56), aBA and aFI (0.5), APOE4 (0.27), aTMT-B (0.23), and sex (0.12). The result indicates that BA and FI are complementary in the prediction of future dementia risk with comparable effect sizes.

### The predictive power of aging biomarkers differs between males and females

Finally, we compared the best-performing aging biomarker model, containing aBA and aFI, for the prediction of 5-year dementia risk for females and males living with MCI separately. Table 2 displays the standardized regression coefficients, model performance, and *p*-values for statistical tests of sex differences in coefficients and performance. Here, the best model of aging biomarkers predicting 5-year DEM risk (sMCI vs pMCI) performed slightly better for females than males (Table 2). The standardized *β*-value for aFI was significantly higher in males (95% CI *β* 0.50 to 0.56) compared with females (*β* 0.42 to 0.50), suggesting a greater risk of DEM progression in men with increasing levels of frailty. Conversely, in the same model, increased aBA (i.e., higher apparent brain age) was a stronger predictor of future DEM risk in females (95% CI *β* 0.53 to 0.60) compared with males (0.32 to 0.37).

**Table 2:** Regression coefficients and model performance for prediction of 5-year dementia risk in female and male subgroups with mild cognitive impairment

	MCI subgroup		
Coefficient/model performance	Female	Male	*p*-value
*b*_Age_	0.21 (0.18–0.24)	0.20 (0.17–0.23)	0.78
*b*_aFI_	0.46 (0.42–0.50)	0.53 (0.50–0.56)	4.60 × 10^−3^
*b*_aBA_	0.57 (0.53–0.60)	0.35 (0.32–0.37)	6.46 × 10^−18^
AUC	0.70 (0.69–0.71)	0.65 (0.65–0.66)	4.98 × 10^−15^

The table shows standardized coefficients, *β*, for chronological age, and the adjusted frailty index (aFI), and brain age (aBA) from logistic regression of 5-year dementia risk in female and male subgroups with mild cognitive impairment (MCI) at baseline as well as model performance for each subgroup. The *p*-values were calculated with a Mann–Whitney *U*-test with 100 bootstrapped train/test splits. Model performance was assessed by the area under the receiver operating characteristic curve (AUC) metric calculated from the full model (including age, aFI, and aBA as predictors) for female and male MCI subgroups, respectively. Coefficient and AUC estimates are reported as mean (95% confidence interval)

### Supplementary analyses

Supplementary Figure 5 shows predictive analyses excluding aFI. Here, we found that aBA outperformed aMA as a predictor for all diagnostic classification tasks, but only slightly. For 5-year DEM risk, CV AUCs were low and comparable (0.56) for both biomarkers. Combining them did not yield further predictive gains for any task.

Supplementary Figure 6 depicts the predictive performance of models where the data-driven FI has been replaced with a standard FI (see “Methods - Functional age” section). While the predictive performance of all models dropped compared with the ones containing the data-driven FI (as shown in Figs. 4 and 5), aBA and aMA still offered marginal value to the diagnostic predictions on top of the standard FI. For the prediction of 5-year DEM risk, the model including age, sex, and the standard FI performed poorly (CV AUC = 0.55). As shown in Supplementary Fig. 6, the overall prediction of prognosis improved when adding BA to the model (BA + FI CV AUC = 0.63), but not MA (stage 2 CV AUC = 0.56). The results indicate that both tested FIs excel in diagnostic prediction and that BA may offer complementary value in prognostic models independently of the type of FI used. Finally, employing the original MA measures instead of the composite aMA did not yield different results for aDunedinPACE (Supplementary Fig. 7) nor aGrimAge2 (Supplementary Fig. 8).

## Discussion

We investigated the predictive value of three aging biomarkers—DNA methylation age (MA), brain age (BA), and frailty index (FI)—for assessing baseline cognitive impairment and future DEM risk in ADNI. Our main findings were as follows: (1) FI outperformed MA and BA and left them redundant in the prediction of the diagnostic group at baseline (e.g., CN vs DEM), (2) BA and FI add complementary and comparable prognostic value beyond routine clinical tests, (3) increases in frailty were particularly strongly associated with adverse 5-year prognosis in males, whereas increases in BA were more strongly associated with adverse prognosis in females, and (4) DunedinPACE and GrimAge2 were associated with poorer memory test results but failed to provide benefit to clinical predictions.

To our best knowledge, this is the first study to test the combined predictive value of MA, BA, and FI in the prediction of dementia-related outcomes. For all diagnostic classification problems, the best model included age, sex, and aFI only. Our best model for distinguishing those with normal cognition from DEM included age, sex, and FI, achieving an AUC of 0.94 in the held-out test set, indicating excellent discrimination. The results confirm the significant relationship between indices of frailty and cognitive impairment previously found in meta-analysis [31]. While both frailty and DEM diagnosis are based on evaluation of function––and thus may overlap––previous research shows that level of frailty is still consistently associated with DEM risk independently of global cognition (e.g., CDR-SB) and neuropsychological test performance (e.g., MMSE) [5, 8, 12]. As such, our results strengthen the notion that frailty should be integral in the assessment of older individuals seeking evaluation for cognitive problems [51].

Studies examining both phenotypic and functional-level aging biomarkers for predicting individuals’ level of cognitive impairment are scarce. Our data suggest that a functional aging biomarker (FI) is superior to upstream aging biomarkers in the prediction of diagnostic status and imply that sophisticated tests of molecular and phenotypic aging might not be necessary at the level of dementia diagnostics, where the level of aging is likely more advanced.

Still, we do acknowledge that our data-driven FI might not be applicable to FIs used in general, which are mostly generated using a manual procedure [49]. As such, we performed supplementary diagnostic predictions using a standard FI generated by others [50] (Supplementary Fig. 5). These results are more in line with the landmark investigation of postmortem neuropathology and frailty by Wallace et al. [52], suggesting some complementary diagnostic merit of neuropathology and frailty combined. Here, the authors investigated the predictive value of a 41-item FI and neuropathological index (NI) counting the number of diverse pathologies on postmortem brain examination on the prediction of normal cognition, MCI, and DEM. Both indices significantly classified CN vs MCI (in-sample AUCs of 0.64 and 0.58 for NI and FI, respectively) and MCI vs DEM (in-sample AUCs of 0.70 and 0.68, respectively). For the same classifications, we obtained similar or superior out-of-sample AUCs using BA and an FI created using the standard procedure by Searle et al. [30]. But as for the main analyses presented above, the added value of BA as a putative non-invasive brain marker for clinical diagnosis was minimal (e.g., CV AUC for MCI vs DEM 0.71 for the standard aFI only and 0.72 for the standard aFI + aBA). Our data would rather suggest that BA is a more promising biomarker in terms of prediction of *future* dementia risk. The unique contribution of aBA in the prediction of 5-year DEM risk reported here, on top of frailty and conventional biomarkers, is novel and strengthens a multidimensional view of DEM [52].

Our findings of sex differences in the prognostic impact of BA and FI on 5-year DEM risk corroborate recent work by Phyo et al. [32]. We found that increased BA had a more adverse impact on 5-year prognosis in females with MCI than males. Conversely, increased frailty had a more negative effect on prognosis in males, aligning with the well-documented “sex-frailty paradox” where frailty has a greater adverse impact on mortality risk in males [53, 54]. The stronger adverse impact of frailty on DEM risk in males is, however, novel (c.fr. [54]) and merits further study.

The results further suggest that higher-level aging biomarkers, theoretically representing more advanced stages of biological age, are more strongly associated with future DEM risk compared to molecular-level metrics. FI and BA outperformed MA in predictive performance on 5-year dementia conversion, while MA provided negligible predictive value. While novel in the context of future DEM risk, the results align with one study of mortality risk. Kim et al. compared a 34-item FI with MA in predicting 4.4-year mortality risk using Cox regressions [55]. Individually, MA strongly predicted mortality, but the relationship became non-significant when accounting for baseline frailty. Conversely, Li et al. found that GrimAge, PhenoAge, and a mortality risk score DNAm algorithm predicted 17-year mortality risk independently of a 34-item FI [34]. Also, while Cole et al. [35] found that combining measures of MA and BA improved mortality risk prediction, we found minimal predictive value in combining the two for the prediction of dementia-related outcomes (Supplemental Fig. 5). The discrepancy in the predictive power of the aging biomarkers in classifying mortality and dementia-related outcomes needs further investigation.

To our knowledge, no prior studies have evaluated FI, GrimAge2, and DunedinPACE for DEM risk. DunedinPACE was associated with future dementia risk in two other MA studies (not including frailty) [9, 10]. Discrepancies in our findings compared with the two previous reports might be partially explained by in-sample testing only and differences in follow-up duration. For example, Thomas et al. reported a 34% increased DEM risk over 14 years for each 1-SD increase in baseline DunedinPACE with Kaplan–Meier curves showing divergence in risk for all DunedinPACE tertiles from 6 to 7 years onwards [10]. These results, along with those of Li et al. [34], suggest the intriguing possibility that different aging biomarkers might play temporally distinct roles in risk prediction across the lifespan, supporting a life course model of DEM risk.

We found an association between DunedinPACE and neuropsychological test results, aligning with Sugden et al. [9], as well as for GrimAge2 in line with [40]. The results suggest age acceleration (as reflected by the chronological age-adjusted DunedinPACE or GrimAge2 scores) may relate to the biological underpinnings of variation in cognitive function. As shown in Fig. 3, our composite aMA-measure based on GrimAge2 and DunedinPACE was also significantly higher in DEM compared with CN, suggesting higher age acceleration on the group level. Conversely, unlike Sugden et al. [9], we did not find group-level differences in DunedinPACE across cognitive status levels (CN, MCI, DEM) in our study which uses the same ADNI data. We believe the discrepancy is due to our study’s smaller, split sample and exclusion criteria, resulting in reduced statistical power.

## Limitations

Our study has limitations. Firstly, our sample size was relatively small, and the nature of sampling in ADNI may limit generalizability to the broader population. Replication in larger, population-level samples such as UK Biobank would be valuable. Furthermore, the sample size was particularly small with respect to participants with MA data, which could explain the lack of predictability deduced from the MA measures and potentially obscuring small effects. Due to the smaller MA dataset, we also prioritized doing a variable selection with FI and BA in a larger stage 1 dataset and then added MA (Fig. 1). This modelling approach, prioritizing predictive efficacy, also limited our ability to directly compare BA and MA, except in the smaller stage 2 data (Supplementary Fig. 5). Secondly and relatedly, we examined molecular aging using a limited set of published MA algorithms. As the field progresses, incorporating novel and more reliable MA measures may offer better predictive value than what we report here. Thirdly, we focused on dementia-related *functional* outcome measures (i.e., clinical diagnosis), not disease-pathology measures such as amyloid or tau. Future studies may evaluate the interplay between aging biomarkers, disease-specific markers, and cognition for a more complete understanding of DEM development and risk (see, e.g., [26]). Finally, we used single-timepoint measurement of the aging biomarkers, which limits the interpretation of temporal dynamics of aging reflected by the markers. For instance, intriguing research by Vidal-Pineiro et al. has shown that BA variation may reflect static, early life factors above current rates of brain aging [56]. While this caveat pertaining to all cross-sectional data is central to the interpretation of the biological underpinnings of the biomarkers, it does not halt their practical use in predictive medicine.

## Conclusion

Our results underscore the potential of combining higher-level biological aging biomarkers in the context of predicting dementia-related outcomes. Future research could explore the biomarkers longitudinally and across longer time frames, tracking changes within individuals to pinpoint inflection points in the aging process and uncover stages of the life span where they provide the most value in risk assessment. The results suggest caution in using or marketing MA algorithms such as DunedinPACE as individual risk markers for shorter-term prognosis or prediction of current cognitive impairment. Additionally, studies integrating disease-specific markers (amyloid, tau) with the present aging-related biomarkers could further our understanding of the interplay between aging and disease.

## Supplementary information

Below is the link to the electronic supplementary material.

Supplementary file1 (PDF 300 KB)

## Data Availability

All data are publicly available (https://ida.loni.usc.edu/). A github-repo is freely available online with the code used to  perform the present analyses (https://github.com/estenhl/frailty).

## Declarations

### Consortium members and affiliations

Alzheimer’s Disease Neuroimaging Initiative Consortium investigators:

Michael Weiner^5^, Paul Aisen^6^, Ronald Petersen^7^, Clifford R. Jack Jr^7^, William Jagust^8^, John Q. Trojanowski^9^, Arthur W. Toga^10^, Laurel Beckett^11^, Robert C. Green^12^, Andrew J. Saykin^13^, John C. Morris^14^, Leslie M. Shaw^9^, Enchi Liu^15^, Tom Montine^16^, Ronald G. Thomas^6^, Michael Donohue^6^, Sarah Walter^6^, Devon Gessert^6^, Tamie Sather^6^, Gus Jiminez^6^, Danielle Harvey^11^, Matthew Bernstein^6^, Nick Fox^17^, Paul Thompson^18^, Norbert Schuff^19^, Charles DeCarli^11^, Bret Borowski^7^, Jeff Gunter^7^, Matt Senjem^7^, Prashanthi Vemuri^7^, David Jones^7^, Kejal Kantarci^7^, Chad Ward^7^, Robert A. Koeppe^20^, Norm Foster^21^, Eric M. Reiman^22^, Kewei Chen^22^, Chet Mathis^23^, Susan Landau^8^, Nigel J. Cairns^24^, Erin Householder^10^, Lisa Taylor Reinwald^24^, Virginia Lee^25^, Magdalena Korecka^25^, Michal Figurski^25^, Karen Crawford^10^, Scott Neu^10^, Tatiana M. Foroud^13^, Steven Potkin^26^, Li Shen^13^, Faber Kelley^13^, Sungeun Kim^13^, Kwangsik Nho^13^, Zaven Kachaturian^27^, Richard Frank^28^, Peter J. Snyder^29^, Susan Molchan^30^, Jeffrey Kaye^31^, Joseph Quinn^31^, Betty Lind^31^, Raina Carter^31^, Sara Dolen^31^, Lon S. Schneider^10^, Sonia Pawluczyk^10^, Mauricio Becerra^10^, Liberty Teodoro^10^, Bryan M. Spann^10^, James Brewer^6^, Helen Vanderswag^6^, Adam Fleisher^6^, Judith L. Heidebrink^20^, Joanne L. Lord^20^, Ronald Petersen^7^, Sara S. Mason^7^, Colleen S. Albers^7^, David Knopman^7^, Kris Johnson^7^, Rachelle S. Doody^32^, Javier Villanueva Meyer^32^, Munir Chowdhury^32^, Susan Rountree^32^, Mimi Dang^32^, Yaakov Stern^33^, Lawrence S. Honig^33^, Karen L. Bell^33^, Beau Ances^24^, Maria Carroll^24^, Sue Leon^24^, Erin Householder^24^, Mark A. Mintun^24^, Stacy Schneider^24^, Angela Oliver^34^, Randall Griffith^34^, David Clark^34^, David Geldmacher^34^, John Brockington^34^, Erik Roberson^34^, Hillel Grossman^35^, Effie Mitsis^35^, Leyla deToledo-Morrell^36^, Raj C. Shah^36^, Ranjan Duara^37^, Daniel Varon^37^, Maria T. Greig^37^, Peggy Roberts^37^, Marilyn Albert^38^, Chiadi Onyike^38^, Daniel D’Agostino II^38^, Stephanie Kielb^38^, James E. Galvin^39^, Dana M. Pogorele^39^, Brittany Cerbone^39^, Christina A. Michel^39^, Henry Rusinek^39^, Mony J. de Leon^39^, Lidia Glodzik^39^, Susan De Santi^39^, P. Murali Doraiswamy^40^, Jeffrey R. Petrella^40^, Terence Z. Wong^40^, Steven E. Arnold^9^, Jason H. Karlawish^9^, David A. Wolk^23^, Charles D. Smith^41^, Greg Jicha^41^, Peter Hardy^41^, Partha Sinha^41^, Elizabeth Oates^41^, Gary Conrad^41^, Oscar L. Lopez^23^, MaryAnn Oakley^23^, Donna M. Simpson^23^, Anton P. Porsteinsson^42^, Bonnie S. Goldstein^42^, Kim Martin^42^, Kelly M. Makino^42^, M. Saleem Ismail^42^, Connie Brand^42^, Ruth A. Mulnard^26^, Gaby Thai^26^, Catherine Mc Adams Ortiz^26^, Kyle Womack^43^, Dana Mathews^43^, Mary Quiceno^43^, Ramon Diaz Arrastia^43^, Richard King^43^, Myron Weiner^43^, Kristen Martin Cook^43^, Michael DeVous^43^, Allan I. Levey^44^, James J. Lah^44^, Janet S. Cellar^44^, Jeffrey M. Burns^45^, Heather S. Anderson^45^, Russell H. Swerdlow^45^, Liana Apostolova^46^, Kathleen Tingus^46^, Ellen Woo^46^, Daniel H. S. Silverman^46^, Po H. Lu^46^, George Bartzokis^46^, Neill R. Graff Radford^47^, Francine Parfitt^47^, Tracy Kendall^47^, Heather Johnson^47^, Martin R. Farlow^13^, Ann Marie Hake^13^, Brandy R. Matthews^13^, Scott Herring^13^, Cynthia Hunt^13^, Christopher H. van Dyck^48^, Richard E. Carson^48^, Martha G. MacAvoy^48^, Howard Chertkow^49^, Howard Bergman^49^, Chris Hosein^49^, Sandra Black^50^, Bojana Stefanovic^50^, Curtis Caldwell^50^, Ging Yuek Robin Hsiung^51^, Howard Feldman^51^, Benita Mudge^51^, Michele Assaly Past^51^, Andrew Kertesz^52^, John Rogers^52^, Dick Trost^52^, Charles Bernick^53^, Donna Munic^53^, Diana Kerwin^54^, Marek Marsel Mesulam^54^, Kristine Lipowski^54^, Chuang Kuo Wu^54^, Nancy Johnson^54^, Carl Sadowsky^55^, Walter Martinez^55^, Teresa Villena^55^, Raymond Scott Turner^56^, Kathleen Johnson^56^, Brigid Reynolds^56^, Reisa A. Sperling^12^, Keith A. Johnson^12^, Gad Marshall^12^, Meghan Frey^12^, Jerome Yesavage^57^, Joy L. Taylor^57^, Barton Lane^57^, Allyson Rosen^57^, Jared Tinklenberg^57^, Marwan N. Sabbagh^58^, Christine M. Belden^58^, Sandra A. Jacobson^58^, Sherye A. Sirrel^58^, Neil Kowall^58^, Ronald Killiany^59^, Andrew E. Budson^59^, Alexander Norbash^59^, Patricia Lynn Johnson^59^, Thomas O. Obisesan^60^, Saba Wolday^60^, Joanne Allard^60^, Alan Lerner^61^, Paula Ogrocki^61^, Leon Hudson^61^, Evan Fletcher^62^, Owen Carmichael^62^, John Olichney^62^, Charles DeCarli^62^, Smita Kittur^63^, Michael Borrie^64^, T. Y. Lee^64^, Rob Bartha^64^, Sterling Johnson^65^, Sanjay Asthana^65^, Cynthia M. Carlsson^65^, Steven G. Potkin^66^, Adrian Preda^66^, Dana Nguyen^66^, Pierre Tariot^22^, Adam Fleisher^22^, Stephanie Reeder^22^, Vernice Bates^67^, Horacio Capote^67^, Michelle Rainka^67^, Douglas W. Scharre^68^, Maria Kataki^68^, Anahita Adeli^68^, Earl A. Zimmerman^69^, Dzintra Celmins^69^, Alice D. Brown^69^, Godfrey D. Pearlson^70^, Karen Blank^70^, Karen Anderson^70^, Robert B. Santulli^71^, Tamar J. Kitzmiller^71^, Eben S. Schwartz^71^, Kaycee M. Sink^72^, Jeff D. Williamson^72^, Pradeep Garg^72^, Franklin Watkins^72^, Brian R. Ott^73^, Henry Querfurth^73^, Geoffrey Tremont^73^, Stephen Salloway^74^, Paul Malloy^74^, Stephen Correia^74^, Howard J. Rosen^12^, Bruce L. Miller^12^, Jacobo Mintzer^75^, Kenneth Spicer^75^, David Bachman^75^, Elizabether Finger^76^, Stephen Pasternak^76^, Irina Rachinsky^76^, John Rogers^76^, Andrew Kertesz^76^, Dick Drost^76^, Nunzio Pomara^77^, Raymundo Hernando^77^, Antero Sarrael^77^, Susan K. Schultz^78^, Laura L. Boles Ponto^78^, Hyungsub Shim^78^, Karen Elizabeth Smith^78^, Norman Relkin^79^, Gloria Chaing^79^, Lisa Raudin^79^, Amanda Smith^80^, Kristin Fargher^80^ & Balebail Ashok Raj^80^

^5^UC San Francisco, San Francisco, CA, USA. ^6^University of California San Diego, San Diego, CA, USA. ^7^Mayo Clinic, Rochester, NY, USA. ^8^UC Berkeley, Berkeley, CA, USA. ^9^University of Pennsylvania, Philadelphia, PA, USA. ^10^University of Southern California, Los Angeles, CA, USA. ^11^Davis, Davis, CA, USA. ^12^Brigham and Women’s Hospital, Harvard Medical School, Boston, MA, USA. ^13^Indiana University, Bloomington, IND, USA. ^14^Janssen Alzheimer Immunotherapy, South San Francisco, CA, USA. ^15^University of Washington, Seattle, WA, USA. ^16^University of London, London, UK. ^17^USC School of Medicine, Los Angeles, CA, USA. ^18^UCSF MRI, San Francisco, CA, USA. ^19^University of Michigan, Ann Arbor, MI, USA. ^20^University of Utah, Salt Lake City, UT, USA. ^21^Banner Alzheimer’s Institute, Phoenix, AZ, USA. ^22^University of Pittsburgh, Pittsburgh, PA, USA. ^23^Washington University St. Louis, St. Louis, MO, USA. ^24^UPenn School of Medicine, Philadelphia, PA, USA. ^25^University of California, Irvine, CA, USA. ^26^Khachaturian, Radebaugh & Associates, Inc and Alzheimer’s Association’s Ronald and Nancy Reagan’s Research Institute, Chicago, IL, USA. ^27^General Electric, Boston, MA, USA. ^28^Brown University, Providence, RI, USA. ^29^National Institute on Aging/National Institutes of Health, Bethesda, MD, USA. ^30^Oregon Health and Science University, Portland, OR, USA. ^31^Baylor College of Medicine, Houston, TX, USA. ^32^Columbia University Medical Center, New York, NY, USA. ^33^University of Alabama Birmingham, Birmingham, MO, USA. ^34^Mount Sinai School of Medicine, New York, NY, USA. ^35^Rush University Medical Center, Chicago, IL, USA. ^36^Wien Center, Vienna, Austria. ^37^Johns Hopkins University, Baltimore, MD, USA. ^38^New York University, New York, NY, USA. ^39^Duke University Medical Center, Durham, NC, USA. ^40^University of Kentucky, Lexington, KY, USA. ^41^University of Rochester Medical Center, Rochester, NY, USA. ^42^University of Texas Southwestern Medical School, Dallas, TX, USA. ^43^Emory University, Atlanta, GA, USA. ^44^University of Kansas, Medical Center, Lawrence, KS, USA. ^45^University of California, Los Angeles, CA USA. ^46^Mayo Clinic, Jacksonville, FL, USA. ^47^Yale University School of Medicine, New Haven, CT, USA. ^48^McGill Univ., Montreal Jewish General Hospital, Montreal, WI, USA. ^49^Sunnybrook Health Sciences, Toronto, ON, Canada. ^50^U.B.C. Clinic for AD & Related Disorders, British Columbia, BC, Canada. ^51^Cognitive Neurology St. Joseph’s, Toronto, ON, Canada. ^52^Cleveland Clinic Lou Ruvo Center for Brain Health, Las Vegas, NV, USA. ^53^Northwestern University, Evanston, IL, USA. ^54^Premiere Research Inst Palm Beach Neurology, West Palm Beach, FL, USA. ^55^Georgetown University Medical Center, Washington, DC, USA. ^56^Stanford University, Santa Clara County, CA, USA. ^57^Banner Sun Health Research Institute, Sun City, AZ, USA. ^58^Boston University, Boston, MA, USA. ^59^Howard University, Washington, DC, USA. ^60^Case Western Reserve University, Cleveland, OH, USA. ^61^University of California, Davis Sacramento, CA, USA. ^62^Neurological Care of CNY, New York, NY, USA. ^63^Parkwood Hospital, Parkwood, CA, USA. ^64^University of Wisconsin, Madison, WI, USA. ^65^University of California, Irvine BIC, Irvine, CA, USA. ^66^Dent Neurologic Institute, Amherst, MA, USA. ^67^Ohio State University, Columbus, OH, USA. ^68^Albany Medical College, Albany, NY, USA. ^69^Hartford Hosp, Olin Neuropsychiatry Research Center, Hartford, CT, USA. ^70^Dartmouth Hitchcock Medical Center, Albany, NY, USA. ^71^Wake Forest University Health Sciences, Winston-Salem, NC, USA. ^72^Rhode Island Hospital, Providence, RI, USA. ^73^Butler Hospital, Providence, RI, USA. ^74^Medical University South Carolina, Charleston, SC, USA. ^75^St. Joseph’s Health Care, Toronto, ON, Canada. ^76^Nathan Kline Institute, Orangeburg, SC, USA. ^77^University of Iowa College of Medicine, Iowa City, IA, USA. ^78^Cornell University, Ithaca, NY, USA. ^79^University of South Florida: USF Health Byrd Alzheimer’s Institute, Tampa, FL, USA.
