# Supplementary material for: Complementary value of molecular, phenotypic, and functional aging biomarkers in dementia prediction
Source: GeroScience. 2024 Oct 24;47(2):2099–118. doi: 10.1007/s11357-024-01376-w (PMC11979055; doi:10.1007/s11357-024-01376-w)
Supplement: Supplementary file 1 — Supplementary file1 (PDF 300 KB) [file 11357_2024_1376_MOESM1_ESM.pdf]

# Supplementary materials for ”Complementary value of molecular, phenotypic and functional aging biomarkers in dementia prediction”

Engvig et al., 2024

## List of Figures


1 Methylation age principal component . . . . . . . . . . . . . . . . . 3
2 Sex-split correlations among potential predictors . . . . . . . . . . . 4
3 Results from the predictive models including methylation age . . . . . 5
4 Confusion matrices of the predictive models . . . . . . . . . . . . 6
5 Performance of models excluding aFI 7
6 Performance of models using standard aFI instead of data-driven . . 8
7 Performance of models using aDunedinPACE instead of aMA . . . . . 9
8 Performance of models using aGrimAge2 instead of aMA . . . . . . . 10

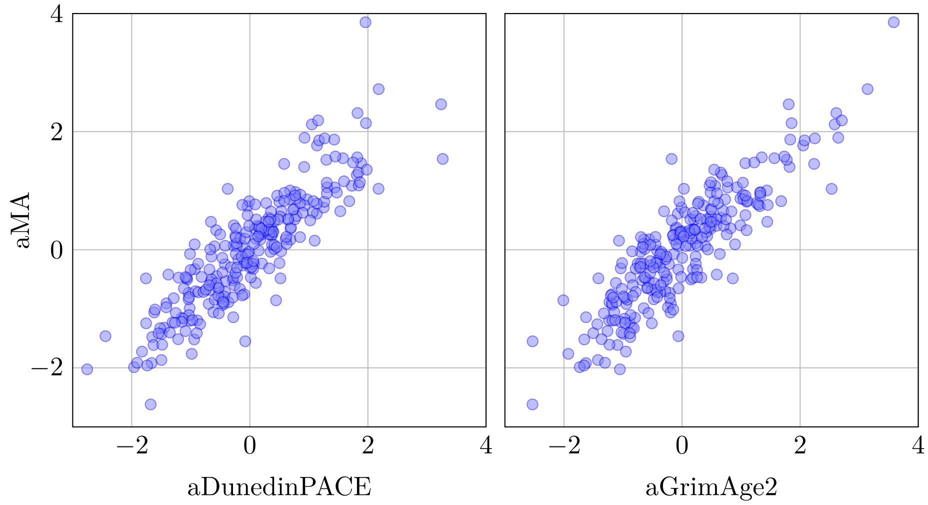


Supplementary Figure 1: Distribution of the original, age- and sex-adjusted, methylation age measures plotted against the first principal component (aMA) from the principal component analysis.

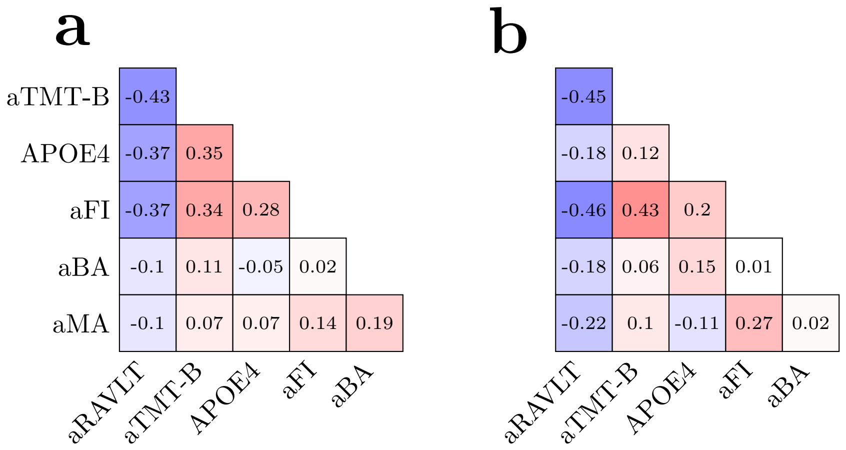


Supplementary Figure 2: Correlations among the clinical variables and aging biomarkers, all age- and sex-adjusted except APOE4, in females (a) and males (b) independently.

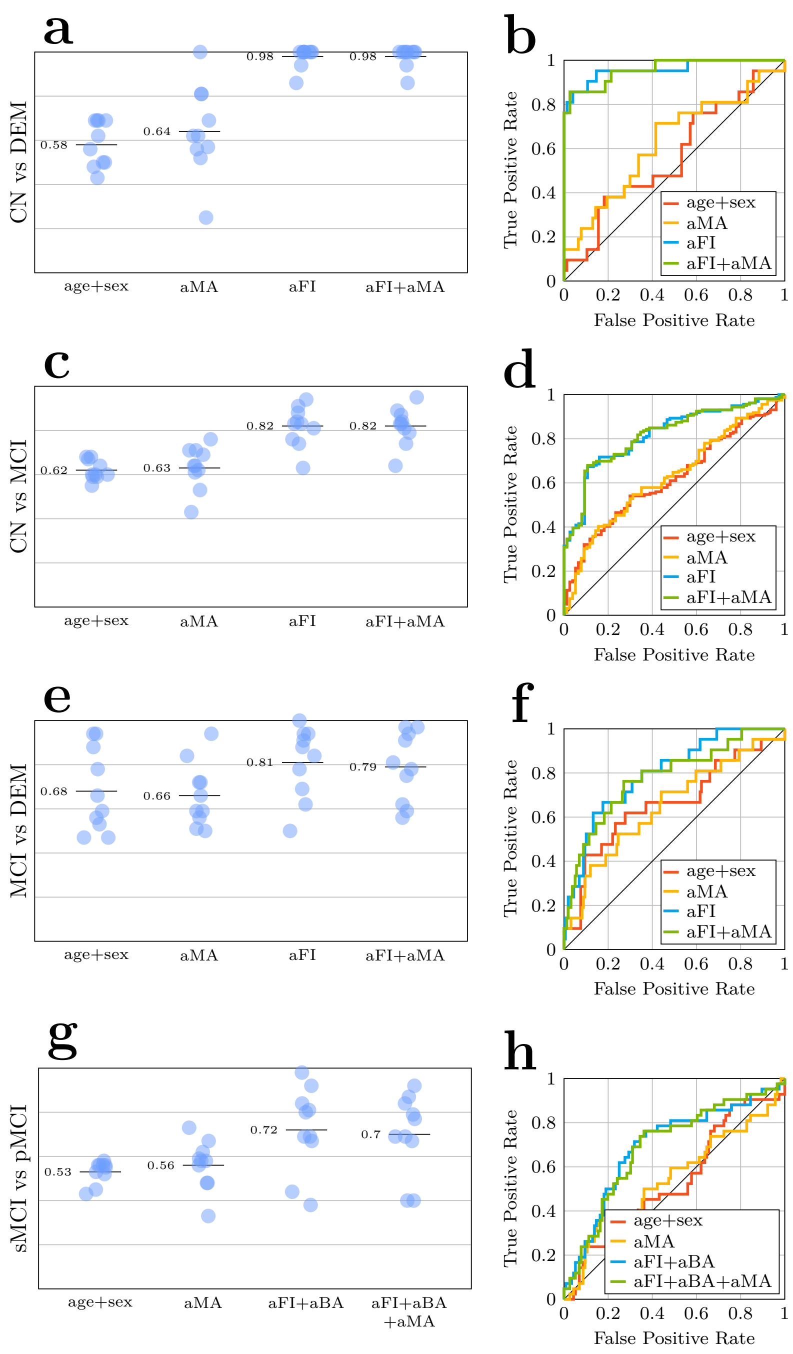


Supplementary Figure 3: Predictive performance of the models including age- and sex-adjusted methylation age (aMA) as predictor along the best subset of predictors from the main analysis, for all four predictive tasks. ROC curves underlying the AUCs can be seen on the right.

**a**

| Observed \ Predicted | 0 | 1 |
|---|---|---|
| 0 | 30 | 8 |
| 1 | 0 | 10 |

**b**

| Observed \ Predicted | 0 | 1 |
|---|---|---|
| 0 | 23 | 15 |
| 1 | 19 | 61 |

**c**

| Observed \ Predicted | 0 | 1 |
|---|---|---|
| 0 | 54 | 26 |
| 1 | 0 | 10 |

**d**

| Observed \ Predicted | 0 | 1 |
|---|---|---|
| 0 | 40 | 19 |
| 1 | 7 | 13 |

Supplementary Figure 4: Confusion matrices for the best performing model in the held-out test data for the four predictive tasks: a) CN vs DEM, b) CN vs MCI, c) MCI vs DEM, and d) sMCI vs pMCI.

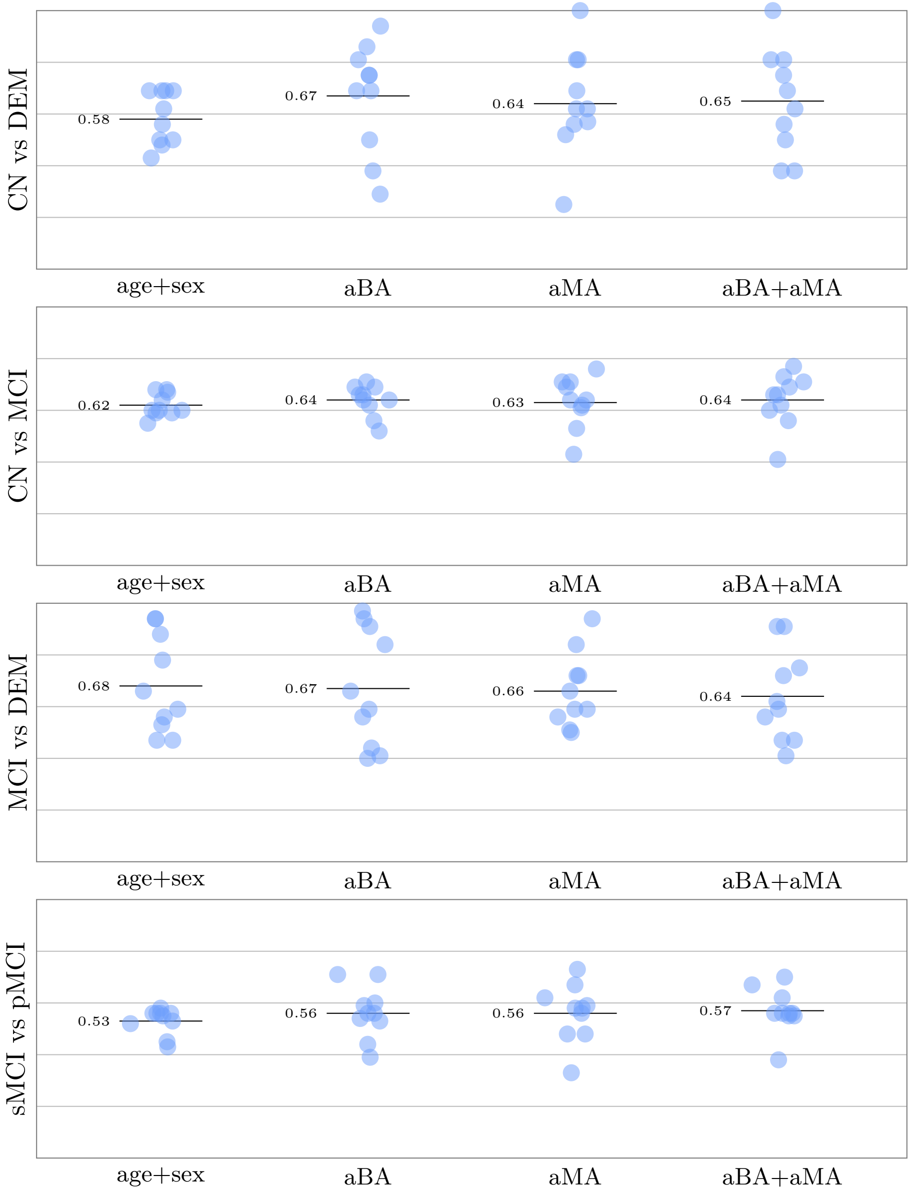


Supplementary Figure 5: Performance of the predictive models excluding aFI as a predictor across all four predictive tasks.

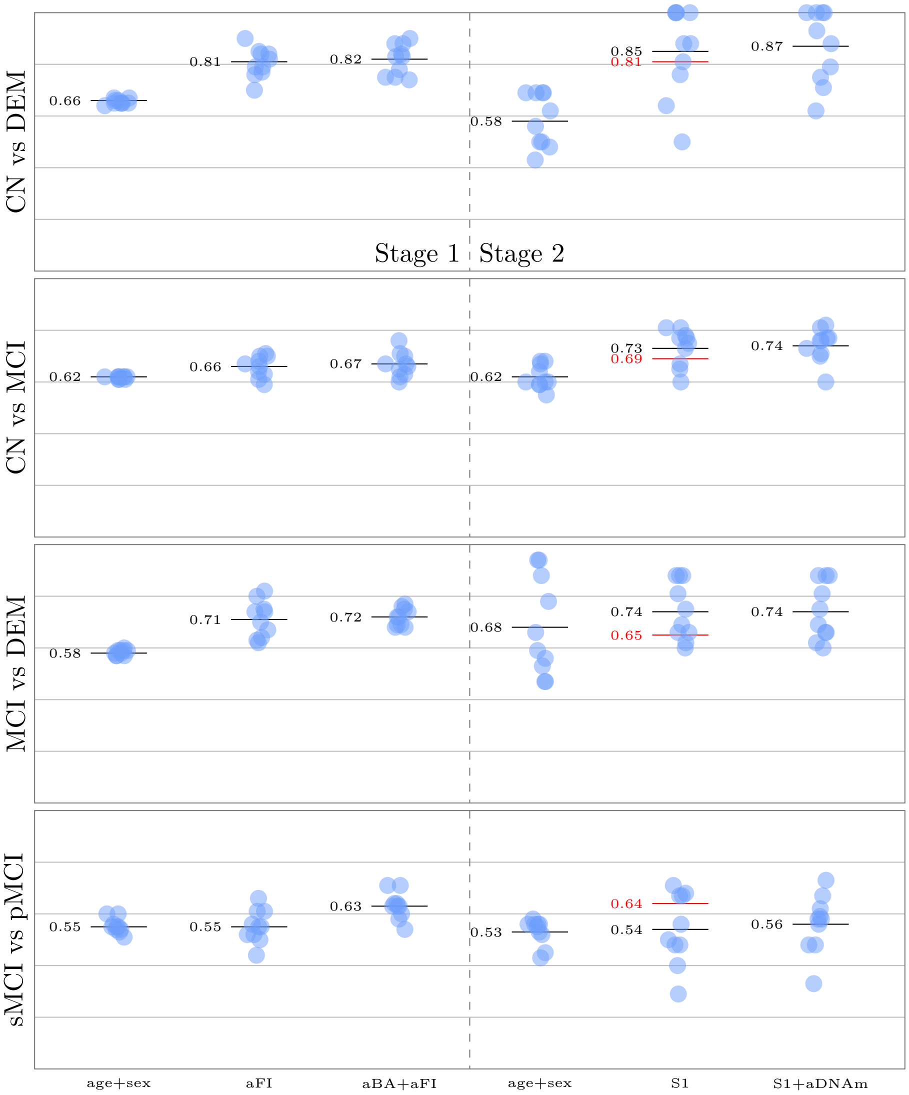


Supplementary Figure 6: Performance of the predictive models using a standard FI measure as a predictor, instead of the data-driven FI used in the main analyses.

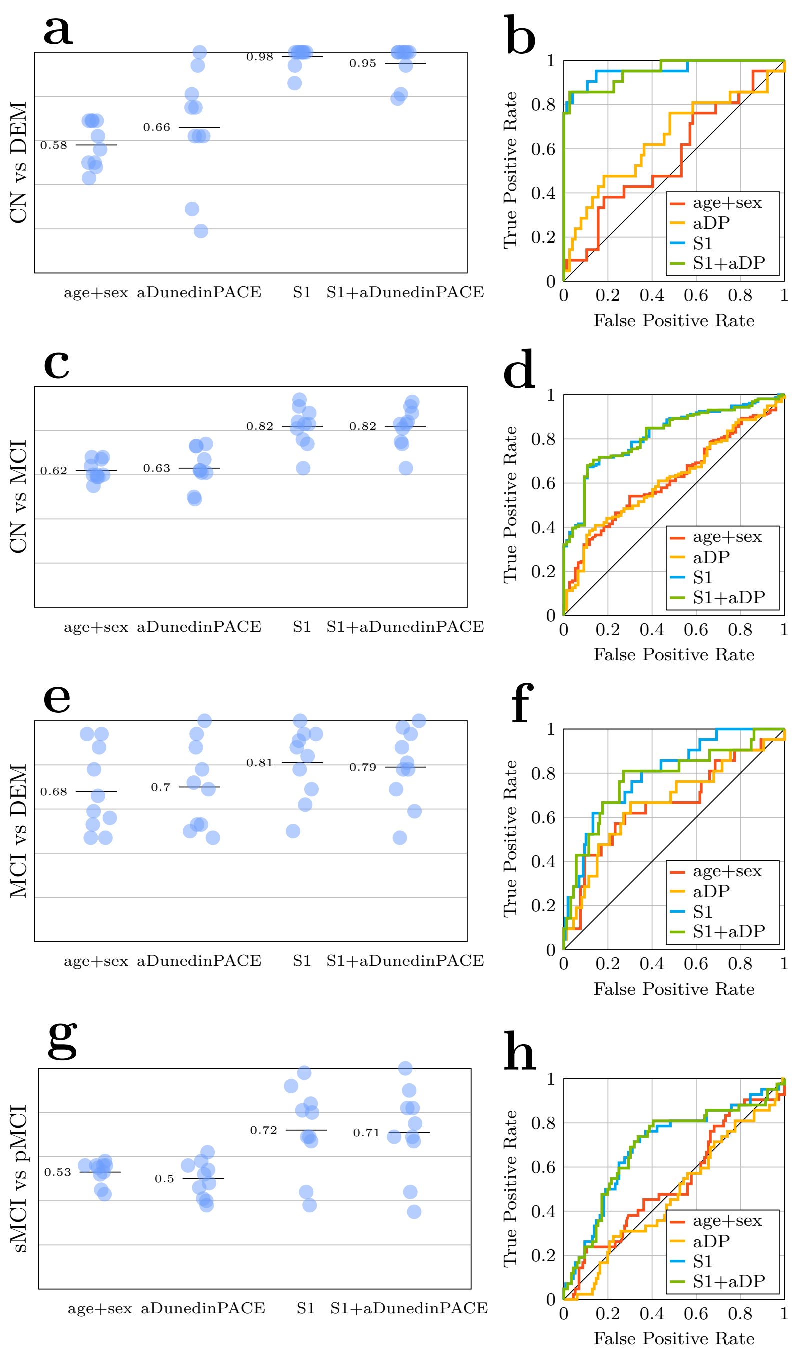


Supplementary Figure 7: Performanceo of the predictive models using the singular aDunedinPACE as a predictor, instead of the composite aMA used in the main analyses.

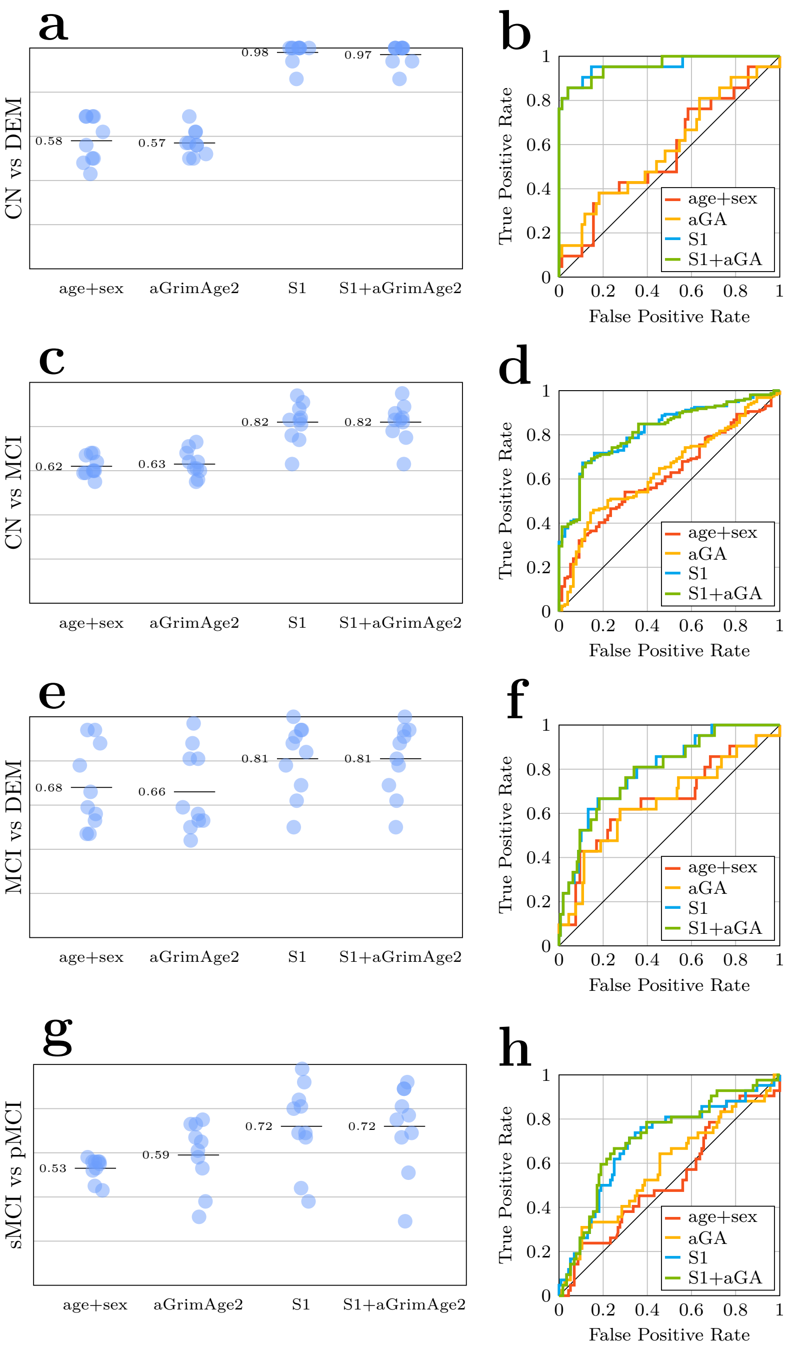


Supplementary Figure 8: Performance of the predictive models using the singular aGrimAge2 as a predictor, instead of the composite aMA used in the main analyses.
